# Heritability of the melatonin synthesis variability in autism spectrum disorders

**DOI:** 10.1038/s41598-017-18016-3

**Published:** 2017-12-18

**Authors:** Marion Benabou, Thomas Rolland, Claire S. Leblond, Gaël A. Millot, Guillaume Huguet, Richard Delorme, Marion Leboyer, Cécile Pagan, Jacques Callebert, Erik Maronde, Thomas Bourgeron

**Affiliations:** 1 Human Genetics and Cognitive Functions Unit, Institut Pasteur, Paris, France; 2 CNRS UMR3571, Genes, Synapses and Cognition, Institut Pasteur, Paris, France; 3 Paris Descartes University, Sorbonne Paris Cité, Paris, France; 4 Paris Diderot University, Sorbonne Paris Cité, Paris, France; 5 Bioinformatics and biostatistics HUB, C3BI, USR 3756 IP CNRS, Institut Pasteur, Paris, France; 6 Child and Adolescent Psychiatry Department, Hôpital Robert Debré, Paris, France; 7 Fondation Fondamental, Créteil, France; 8 Psychiatry Department, Hôpital Henri Mondor-Albert Chenevier, AP-HP, Université Paris Est, Créteil, France; 9 INSERM U955, Translational Psychiatry, Paris-Est University, Créteil, France; 10 Service de Biochimie et Biologie Moléculaire, INSERM U942, Hôpital Lariboisière, APHP, Paris, France; 11 Institute for Cellular and Molecular Anatomy, Dr. Senckenbergische Anatomie, Goethe-University, Frankfurt, Germany

## Abstract

Autism Spectrum Disorders (ASD) are heterogeneous neurodevelopmental disorders with a complex genetic architecture. They are characterized by impaired social communication, stereotyped behaviors and restricted interests and are frequently associated with comorbidities such as intellectual disability, epilepsy and severe sleep disorders. Hyperserotonemia and low melatonin levels are among the most replicated endophenotypes reported in ASD, but their genetic causes remain largely unknown. Based on the biochemical profile of 717 individuals including 213 children with ASD, 128 unaffected siblings and 376 parents and other relatives, we estimated the heritability of whole-blood serotonin, platelet N-acetylserotonin (NAS) and plasma melatonin levels, as well as the two enzymes arylalkylamine N-acetyltransferase (AANAT) and acetylserotonin O-methyltransferase (ASMT) activities measured in platelets. Overall, heritability was higher for NAS (0.72 ± 0.091) and ASMT (0.59 ± 0.097) compared with serotonin (0.31 ± 0.078), AANAT (0.34 ± 0.077) and melatonin (0.22 ± 0.071). Bivariate analyses showed high phenotypic and genetic correlations between traits of the second step of the metabolic pathway (NAS, ASMT and melatonin) indicating the contribution of shared genetic factors. A better knowledge of the heritability of the melatonin synthesis variability constitutes an important step to identify the factors that perturb this pathway in individuals with ASD.

## Introduction

Autism Spectrum Disorders (ASD) are complex neurodevelopmental disorders characterized by deficits in social communication/interaction as well as restricted interests and repetitive patterns of behaviors. Although ASD are highly heritable disorders^1–3^, they are multifactorial and clinically heterogeneous and the ASD-risk genes remain largely unknown. Large-scale genetic studies revealed a complex genetic architecture, involving combined effects of multiple low risk common variants, rare and *de novo* deleterious mutations^4^. Endophenotypes are measurable markers with potentially reduced genetic heterogeneity compared to the disease itself and they can be used to overcome the genetic and phenotypic heterogeneity. They facilitate the stratification of patients into more homogeneous subgroups^5^, increase the power of quantitative genetic analyses to detect variants, genes or pathways associated with the disease of interest and they reveal underlying biological mechanisms.

Hyperserotonemia is one of the most replicated endophenotypes in ASD^6–10^. A recent meta-analysis including 739 patients and 868 controls estimated an elevated whole-blood serotonin in 28.3% of the patients with ASD compared to 5% of controls^11^. Low melatonin levels in urine, plasma and pineal gland have also been described in ASD individuals^10,12–15^. Melatonin derives from serotonin, which is successively converted into N-acetylserotonin (NAS) and melatonin by the enzymes arylalkylamine N-acetyltransferase (AANAT, EC: 2.3.1.87) and acetylserotonin O-methyltransferase (ASMT, EC: 2.1.1.4). Melatonin biosynthesis essentially occurs in the pineal gland, following a marked circadian rhythm with a maximal secretion at night. A global disruption of this pathway was observed in patients compared to relatives and controls, including hyperserotonemia, deficits in AANAT and ASMT platelet activity, increased platelet NAS and melatonin deficit^10,15^. These alterations were also observed to a lesser extent, in the relatives of patients with ASD, compared to controls^10,15^, but familial correlations and heritability have never been assessed for both steps of the melatonin synthesis pathway in families with ASD.

Serotonin is involved in a wide range of central processes, such as brain development, emotion, learning, memory or cognitive functions^16,17^. NAS is an agonist of melatonin receptors and activates TrkB in mice, a receptor of the brain-derived neurotrophic factor (BDNF), in a selective and circadian manner^18,19^. Melatonin is a pleiotropic neuroendocrine molecule essential for synchronizing circadian and seasonal rhythms, as well as sleep/wake cycles, but also displays antioxidant, neuroprotective, or immunomodulatory effects^20–22^. Biochemical alterations of this pathway could thus be related to the core symptoms of autism or the comorbidities such as cognitive problems, epilepsy, sleep and gastrointestinal disorders observed in ASD^17,23–25^.

In order to identify shared underlying genetic factors between an endophenotype and a disease, the endophenotype must be heritable with either high broad sense heritability (H^2^), defined as the ratio of total genetic variance (additive, dominance and epistasis) to phenotypic variance, or high narrow sense heritability (h^2^), reflecting only the additive part (the average effect of the alleles on the trait) of the genetic contribution to the phenotypic variance (Supplementary Note). Several studies in humans and in animal models have demonstrated relatively high heritability for serotonin^26–28^ and melatonin^29–32^. In humans, serotonin narrow sense heritability estimates range from 0.2 to 0.51^26,27,33^ and urinary melatonin narrow sense heritability estimate was 0.53 in families with acute intermittent porphyria^32^. To our knowledge, NAS, AANAT and ASMT heritability have never been investigated. In this study, we estimated the narrow sense heritability of all five quantitative traits (whole blood serotonin, platelet AANAT and ASMT activities, platelet NAS and morning plasma melatonin) in 717 individuals including 213 children with ASD, 364 parents, 128 unaffected siblings and 12 other relatives (Supplementary Fig. S1)^10,15^. We also evaluated the correlations of genetic and environmental factors between pairs of traits. The assessment of additive factors contribution to the inter-individual phenotypic variability should provide crucial information for further quantitative genetic investigations of this pathway in families with ASD.

## Results

### Biochemical ascertainment of the melatonin synthesis pathway

The study sample included 717 participants (182 patients with ASD, 364 parents, 128 unaffected siblings, 31 affected siblings and 12 other relatives) with a mean age of 31.3 years and 59.3% males (425/717). As frequently observed in ASD^34,35^, the sex ratio for the patients in our cohort was one female for 4.5 males (39 females and 174 males) (Table 1), whereas the number of males and females was similar in the unaffected siblings (M/F = 62/66 = 0.94). As previously reported for this cohort^10,15^, there were significant biochemical differences between patients and their relatives (parents or unaffected siblings) for each biochemical parameter: hyperserotonemia, increased platelet NAS, and deficit in platelet ASMT, platelet AANAT and plasma melatonin (Table 1).

**Table 1:** Characteristics of the studied population including 185 families with ASD investigated for the melatonin synthesis pathway biochemical traits.

	All (415 males, 290 females)		Affected (A) (174 males, 39 females)		Unaffected (U) (62 males, 66 females)		Parents (P) (179 males, 185 females)		Wilcoxon rank-sum test		
	n	M (SD)	n	M (SD)	n	M (SD)	n	M (SD)	P_A/U_	P_A/P_	P_U/P_
Age	705	31.1 (18.6)	213	14.4 (9.1)	128	14.8 (8.0)	364	46.5 (9.5)	0.33		
Serotonin	621	491.1 (319.7)	189	645.8 (407.6)	111	433.5 (252.2)	321	419.9 (241.2)	**9.4 × 10** ^**−6**^	**4.4 × 10** ^**−10**^	0.63
AANAT	486	3.9 (0.9)	155	3.6 (0.9)	72	4.0 (0.8)	259	4.0 (0.8)	**1.1 × 10** ^**−3**^	**2.8 × 10** ^**−6**^	0.87
NAS	348	36.9 (15.0)	107	44.3 (15.9)	56	35.0 (13.3)	185	33.2 (13.3)	**6.4 × 10** ^**−5**^	**3.2 × 10** ^**−10**^	0.41
ASMT	381	1.1 (0.7)	118	0.8 (0.6)	56	1.2 (0.7)	207	1.2 (0.8)	**1.6 × 10** ^**−4**^	**2.3 × 10** ^**−10**^	0.38
Melatonin	521	0.12 (0.08)	157	0.10 (0.071)	100	0.14 (0.08)	264	0.14 (0.08)	**3.6 × 10** ^**−8**^	**2.4 × 10** ^**−11**^	0.70

Affected include probands with ASD and affected siblings. Unaffected include unaffected siblings. The 12 other relatives (affected parents, grandparents, and uncles) are not included in this table. Significant p-values after Bonferroni correction are indicated in bold: 16 tests were performed including 15 critical tests (age was not relevant) p-values < 3.33 × 10^−3^ (0.05/15) were considered as significant. Blood serotonin (nM); platelet AANAT and ASMT activities (pmol/10^9^ platelets/30 min); platelet NAS (nmol/10^9^ platelets); melatonin (nM); age (years); M, Mean; SD, Standard Deviation; n, numbers of subjects; P_A/U_, p-value of the test comparing affected children to unaffected children; P_A/P_, p-value of the test comparing affected children to the parents; P_U/P_, p-value of the test comparing unaffected children to the parents.

**Figure 1:**
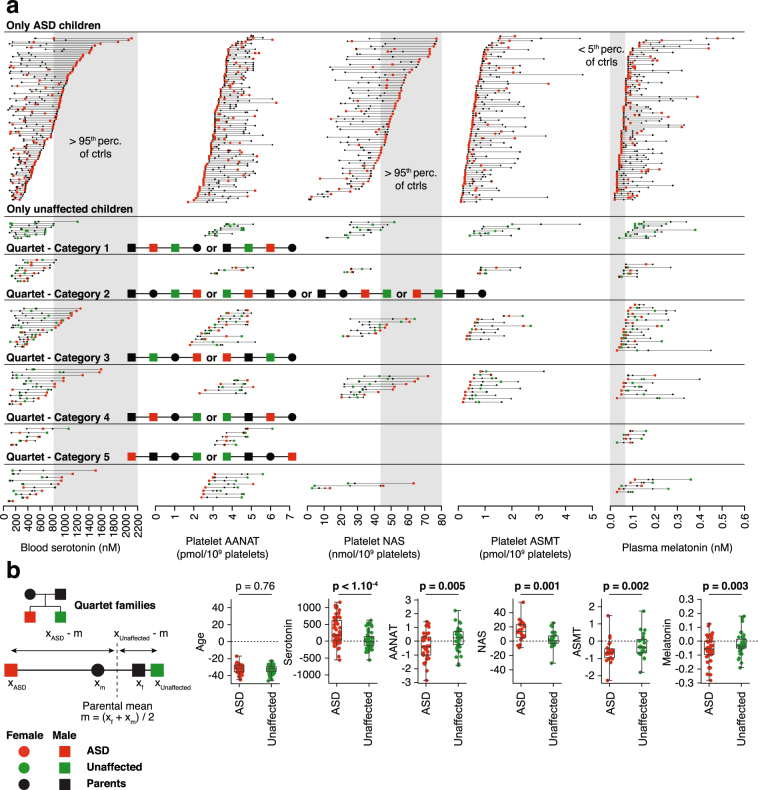
Biochemical values of molecules of the melatonin synthesis pathway. (**a**) Families studied in this work are divided into those with only ASD children, those with only unaffected children, and quartet families. Quartet families are then separated between those with 1) both children values within parental values, 2) both children values higher or lower than parental values, one children value within parental values and the other 3) ASD or 4) unaffected children value higher or lower, and 5) both children values outside the range of parental values but on opposite directions. Grey areas indicate pathological ranges based on previous studies in control populations (95^th^ percentile of the controls for serotonin and NAS, 5^th^ percentile for melatonin)^10^. All families are ordered according to the highest (serotonin and NAS) or lowest (AANAT, ASMT and melatonin) value observed for this family in the trait studied. (**b**) Quartet families selected are those for which the sibling and the two parents of the ASD proband are unaffected. For each trait monitored, the average of the two parental values was computed, and the distance from this average was measured for the two children. Distances of the ASD and the unaffected children are compared based on a Wilcoxon sign test and significance assessed after Bonferroni correction (6 tests were performed, p-values < 8.3 × 10^−3^ (0.05/6) were considered as significant).

Regarding the distribution of the biochemical traits within pedigrees, different categories of families could be determined (Fig. 1a). We observed that for some families, affected and/or unaffected children displayed values within the range defined by their corresponding parental values, while in other families, children biochemical values were outside this range. In most cases, children with ASD had the most extreme values compared to unaffected children. To quantify the difference between parents and affected or unaffected children, we used quartet families including one affected child and one unaffected child (Fig. 1b). For each trait, the distance between children and average parental values were compared. We observed that affected children values were significantly more distant (P < 0.005) from their corresponding average parental values than unaffected children. We therefore performed detailed familial correlation and heritability analyses of each step of the melatonin synthesis pathway.

### Heritability of the melatonin synthesis variability

We first calculated the correlation coefficients for parent-offspring (P-O), sibling-sibling (S-S) and father-mother (F-M) pairs (Table 2). When all individuals of the families were taken into account (affected and unaffected), the P-O correlation coefficients were significantly different from 0 for all traits after Bonferroni correction for multiple testing, except for melatonin (uncorrected p-value = 0.027), and the correlation was particularly high for NAS compared with other parameters (NAS: 0.38; ASMT: 0.28; AANAT: 0.23; serotonin: 0.15, and melatonin: 0.13) (Table 2). No S-S correlations remained significant after Bonferroni correction. Interestingly, F-M correlations were significant for AANAT and melatonin.

**Table 2:** Familial correlations of the biochemical traits in the studied cohort.

Data	Group	Parent-offspring correlation (P-O)		Sibling-sibling correlation (S-S)				Father-mother correlation (F-M)		
		N pairs	Correlation (SE)	P-value	N pairs	Correlation (SE)	P-value	*N pairs	Correlation (SE)	P-value
Serotonin	^b^All	606	0.15 (0.043)	0.0005	187	0.13 (0.080)	0.11	165	0.058 (0.078)	0.46
	^a^ASD	382	0.12 (0.054)	0.029	30	0.094 (0.21)	0.66	162	0.066 (0.079)	0.41
	^a^Unaffected	216	0.20 (0.070)	0.0063	35	0.18 (0.32)	0.59	84	0.094 (0.11)	0.40
AANAT	^b^All	460	0.23 (0.057)	**0.0001**	138	0.26 (0.11)	0.022	133	0.34 (0.077)	**0.0001**
	^b^ASD	314	0.22 (0.064)	**0.0007**	25	0.19 (0.21)	0.38	133	0.34 (0.077)	**<0.0001**
	^c^Unaffected	140	0.36 (0.094)	**0.0006**	26	0.099 (0.22)	0.65	54	0.59 (0.091)	**<0.0001**
NAS	^a^All	328	0.38 (0.056)	**<0.0001**	101	0.21 (0.12)	0.11	96	0.13 (0.10)	0.20
	^a^ASD	214	0.40 (0.063)	**<0.0001**	12	0.42 (0.25)	0.13	95	0.16 (0.10)	0.13
	^c^Unaffected	102	0.32 (0.11)	0.0077	20	0.63 (0.20)	0.014	38	0.080 (0.17)	0.64
ASMT	^a^All	354	0.28 (0.053)	**<0.0001**	104	0.23 (0.12)	0.071	107	−0.089 (0.097)	0.37
	^a^ASD	240	0.24 (0.061)	**0.0001**	13	0.59 (0.19)	0.013	107	−0.082 (0.097)	0.4019
	^a^Unaffected	108	0.32 (0.090)	0.0012	21	0.51 (0.23)	0.058	40	−0.21 (0.16)	0.20
Melatonin	^a^All	518	0.13 (0.057)	0.027	173	0.27 (0.088)	0.0035	136	0.30 (0.078)	**0.0003**
	^b^ASD	318	0.055 (0.072)	0.45	28	0.23 (0.21)	0.29	132	0.31 (0.079)	**0.0003**
	^a^Unaffected	192	0.21 (0.090)	0.025	35	0.62 (0.16)	0.0023	73	0.20 (0.11)	0.10

Significant p-values after Bonferroni correction are indicated in bold: 45 tests were performed, p-values < 1.11 × 10^−3^ (0.05/45) were considered as significant. N pairs, number of relative pairs included in the analysis; SE, Standard Error; ^a^age was included in the model as a covariate; ^b^age and sex were included in the model as covariates; ^c^no covariates were included in the model. *Number of father-mother pairs (F-M): parents included in the analyses using all family members and in stratified analyses as described in Supplementary Table S4.

**Figure 2:**
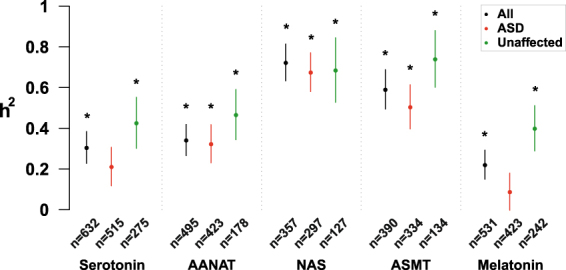
Narrow sense heritability estimates (h^2^) of the biochemical traits associated with the melatonin synthesis pathway. Heritability was calculated using the variance component analysis with maximum likelihood estimation method. When the effect of the covariates (age, sex) was significant (P < 0.1), they were included in the model. The significance of heritability estimates is assessed using a loglikelihood ratio test that compares the polygenic model to a sporadic model in which the additive genetic effect is constrained to zero. n, number of participants; dots, h^2^ estimates; error bars, standard errors. *Bonferroni corrected P-value < 0.05 (15 tests were performed, p-values < 3.3 × 10^−3^ (0.05/15) were considered as significant).

We then estimated the heritability of each trait. When all individuals were taken into account, the narrow sense estimates of heritability (reflecting additive effects) were all significant (Bonferroni corrected p-value < 0.05; Fig. 2 and Supplementary Table S1). The heritability estimates ranged from 0.22 for melatonin to 0.72 for NAS (serotonin: 0.31; AANAT: 0.34; NAS: 0.72; ASMT: 0.59; melatonin: 0.22) providing support for a significant genetic contribution to the melatonin synthesis variability. Proportions of variance due to all covariates included were relatively low, ranging from 0.029 to 0.072 (Supplementary Table S2). When the sample was stratified by ASD status, all heritability estimates tended to be higher for unaffected children than for children with ASD (Fig. 2 and Supplementary Table S1). For example, both serotonin and melatonin heritability estimates were not significant for children with ASD, after Bonferroni correction for multiple testing.

**Figure 3:**
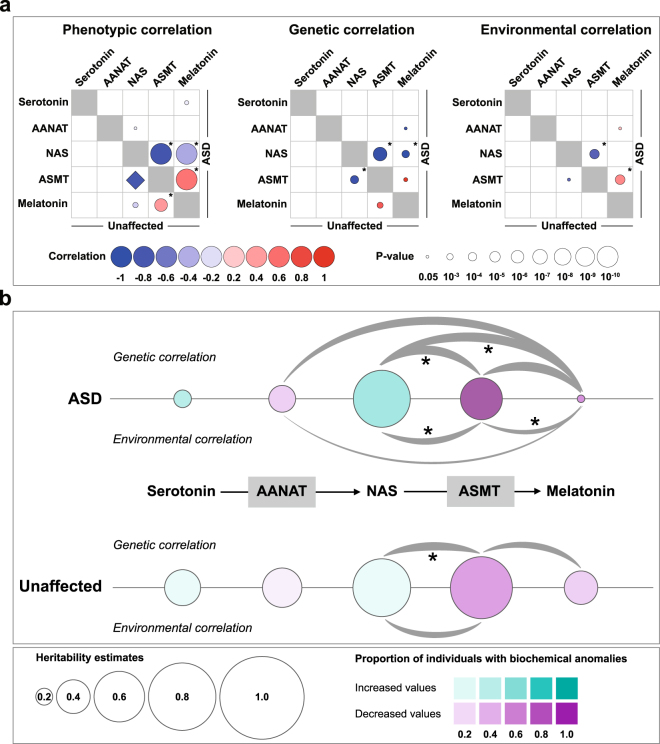
Heritability analysis of the melatonin synthesis pathway in families with ASD. **(a)** Bivariate heritability analyses. Pairwise phenotypic correlations (ρ_P_), additive genetic correlations (ρ_G_) and shared environmental correlations (ρ_E_). Correlations calculated in patients with ASD are on the upper diagonal; correlations calculated in unaffected siblings are on the lower diagonal. P-values test the significance of the difference of ρ_P_, ρ_G_ and ρ_E_ from zero. The diamond shape used for the phenotypic correlation between ASMT and NAS in unaffected individuals means that the p-value could not be tested according to SOLAR. *Significant p-values after Bonferroni correction: 60 tests were performed, p-values < 8.33 × 10^−4^ (0.05/60) were considered as significant. **(b)** Overview of heritability analyses results for the biochemical traits associated with the melatonin synthesis pathway in patients with ASD and in unaffected individuals. Circle sizes represent heritability estimates. Circle colors represent the proportion of patients or unaffected siblings with biochemical values >95^th^ percentile of controls in purple and <5^th^ percentile of controls in green, as previously published^13,19^. Grey curved lines represent environmental and genetic correlations with nominal P-values < 0.05 and their thickness is proportional to correlation values. *Significant correlations after Bonferroni correction.

To investigate the relationship between each pair of traits and evaluate the proportion of heritability of one trait that is attributable to the other trait, we calculated bivariate trait correlation coefficients for ASD and unaffected children separately (Fig. 3a). In both groups, we observed phenotypic correlations ρ_P_ > 0.4 for ASMT-melatonin, ρ_P_ < −0.8 for NAS-ASMT and a ρ_P_ < −0.2 for NAS-melatonin. These results confirmed a high correlation between the three parameters of the last step of the melatonin biosynthesis pathway (NAS, ASMT and melatonin), as previously reported^15^. When only children with ASD were included, these phenotypic correlations were significantly different from 0 (NAS-ASMT: ρ_P_ = −0.84; ASMT-melatonin: ρ_P_ = 0.57; NAS-melatonin: ρ_P_ = −0.42) (Fig. 3a). For unaffected children, only ASMT-melatonin phenotypic correlation remained significant after Bonferroni correction (ASMT-melatonin: ρ_P_ = 0.42). Regarding genetic correlations, two negative genetic correlations were significantly different from 0 in children with ASD (NAS-ASMT: ρ_G_ = −0.96; NAS-melatonin: ρ_G_ = −1.0) and in unaffected children, only one genetic correlation was significant (NAS-ASMT: ρ_G_ = −0.90) (Fig. 3a). These genetic correlations were not significantly different from 1 or −1. There were also significant common environmental influences for NAS-ASMT (ρ_E_ = −0.70) and for ASMT-melatonin (ρ_E_ = 0.48) in children with ASD (Fig. 3a). In conclusion, these results showed an overlap of additive genetic effects as well as shared environmental factors influencing phenotypic variability in families with ASD, particularly for NAS, ASMT and melatonin (Fig. 3b).

## Discussion

Very few heritability estimates for serotonin and melatonin are available for humans in the literature (Table 3) and this study is the first to characterize the heritability of the melatonin synthesis variability in a large sample of families with ASD. We first calculated familial correlations of the five traits (serotonin, AANAT, NAS, ASMT and melatonin). Using all family members, we observed significant P-O correlations except for melatonin, but no S-S correlations remained significant after correction for multiple testing, probably due to the relatively small number of S-S pairs compared to P-O pairs. Unexpectedly, we also observed significant correlations between fathers and mothers for AANAT and melatonin. AANAT activity and melatonin production display marked circadian variations in the pineal gland^15,36^. These processes are closely related to light-dark cycle^37–39^ and their regulation might depend on environmental factors, such as sleep-wake cycle and artificial light exposure. Thus, synchronization could be observed between parents that share daily habits and life rhythms.

**Table 3:** Review of the literature on serotonin and melatonin heritability estimates in humans.

Trait	Study	Heritability estimates and main findings	N	Subjects	Tissue	Method
Serotonin	Abney *et al*.^27^	h^2^ = 0.51 (SE: 0.11) H^2^ = 1.0 (SE: 0.25)	567	Hutterite families, large pedigrees	Blood	Maximum-likelihood with variance component model. Best-fitting model: ADE^58^
	Coutinho *et al*.^59^	H^2^ = 0.64	327	Trio families with ASD	Platelet	Maximum-likelihood with variance component model. Polygenic model (QTDT^60^)
	Shin *et al*.^26^	h^2^ = 0.331 (95% CI: 0.168, 0.426)	866 MZ pairs 878 DZ pairs	TwinsUK cohort	Blood	Maximum-likelihood with variance component model. ACE model (OpenMx^61^)
	Rhee *et al*.^33^	h^2^ ∼ 0.2	2,076	Framingham Heart Study Offspring cohort families	Blood	Maximum-likelihood with variance component model. Additive genetic model (SOLAR^54^)
	This study	h^2^ = 0.31 (SE: 0.08)	632	PARIS cohort families (all individuals)	Blood	Maximum-likelihood with variance component model. Additive genetic model (SOLAR^54^)
		h^2^ = 0.21 (SE: 0.09)	515	PARIS cohort families (parents and affected children)		
		h^2^ = 0.43 (SE: 0.13)	275	PARIS cohort families (parents and unaffected children)		
Melatonin	Wetterberg *et al*.^32^	h^2^ = 0.53	107	Swedish families with acute intermittent porphyria	Urine	Morton and MacLean mixed model for complex segregation analysis^62,63^
	Hallam *et al*.^31^	r_MZ_ = 0.928 r_DZ_ = 0.867	6 MZ pairs 11 DZ pairs	Australian Twin Registry	Plasma	Monozygotic and dizygotic twin correlations
	This study	h^2^ = 0.22 (SE: 0.07)	531	PARIS cohort families (all individuals)	Plasma	Maximum-likelihood with variance component model. Additive genetic model (SOLAR^54^)
		h^2^ = 0.09 (SE: 0.09)	423	PARIS cohort families (parents and affected children)		
		h^2^ = 0.40 (SE: 0.11)	242	PARIS cohort families (parents and unaffected children)		

N, number of subjects; h^2^, narrow sense heritability; H^2^, broad sense heritability; SE, standard error; MZ, monozygotic twins; DZ, dizygotic twins; A, additive; D, dominant; E, unique environment; C, common environment; r_MZ_, monozygotic twin correlation; r_DZ_, dizygotic twin correlation.

We then estimated the heritability of the five traits. In the literature, the first estimate of blood serotonin heritability was obtained by Abney *et al*.^27^ in an inbred founder population of Hutterite families. They found respectively 0.51 and 1.0 for narrow and broad sense heritability, suggesting a very strong genetic influence with both additive and dominance components. Our estimation of serotonin narrow sense heritability was lower (0.31) than the one reported by Abney *et al*., but very similar to the one obtained in a large cohort of 866 monozygotic and 878 dizygotic unaffected twin pairs (0.33; Table 3)^26^. For melatonin, our estimation of the heritability (0.22) ranged between two previous estimates^31,32^. Wetterberg *et al*. (1983) estimated melatonin heritability to 0.53 in 107 families with acute intermittent porphyria^32^. In contrast, a twin study measuring late night plasma melatonin in nine monozygotic and 11 dizygotic twin pairs, reported a high monozygotic correlation (*r*_MZ_ = 0.928) and a high dizygotic correlation (*r*_DZ_ = 0.867)^31^. Using these correlations and the Falconer’s formula (H^2^ = 2(rMZ - rDZ)), the heritability for melatonin would be 2(0.928-0.867) = 0.12.

All the five traits studied here are not expected to be independent since they belong to the same metabolic pathway. Using a bivariate approach, we estimated pairwise phenotypic, genetic and environmental correlations in samples including only children with ASD or only unaffected children. In both groups, phenotypic correlations appeared to be particularly high for the last step of melatonin biosynthesis pathway (NAS, ASMT and melatonin) (Fig. 3b). Therefore, considering these traits as dependent might be useful for further association studies exploring variants associated with these phenotypes. Interestingly, although ASMT and NAS were phenotypically correlated with melatonin, their heritability differed, particularly in children with ASD (high for ASMT and NAS, low for melatonin). In children with ASD, this discrepancy could be explained for ASMT and melatonin by the fact that phenotypic correlation between these traits was mainly attributable to shared environment while genetic factors seemed to be more divergent (Fig. 3a). However, for NAS and melatonin, genetic correlation was high and environmental correlation was not significant. These results suggest that even if there are probably common genetic mechanisms in the regulation of these three traits, environmental factors such as light exposure or drugs interacting with cytochromes, might act differentially on NAS and melatonin levels.

There are several limitations of this study such as the absence of data on circadian rhythm. However, previous studies showed that the deficit in melatonin in ASD was also observed in blood and urine samples collected during the night^12,13,40^. Another limitation is the absence of analyses in the pineal gland. Finally, our estimations for unaffected children might not reflect the heritability the melatonin synthesis variability in the general population. Nevertheless, for serotonin and melatonin, our heritability estimates are very similar from those obtained in previous studies (Table 3)^26,27,32,33^.

In summary, our results revealed that the resemblance between unaffected children and parents seems to be mostly due to additive genetic effects, while patients resemblance to their parents, which was lower, probably includes additional factors such as *de novo* events, dominant effects, or non-shared environmental influences related to ASD condition. Another interesting finding is that NAS displayed the highest heritability estimate within the melatonin synthesis pathway. In contrast to serotonin and melatonin, NAS received much less interest from the scientific and clinical community^10,41^. This is unfortunate, especially since NAS could have its own biological functions, such as TrkB activation, immunomodulation or analgesia^19,41–44^. We thus propose to use NAS, in addition to serotonin and melatonin, as a suitable endophenotype for further quantitative genetic studies in ASD. Further studies using larger study samples and molecular genetic analyses such as genome-wide association studies are now required to identify the variants involved in melatonin synthesis variability in the general population and in patients suffering from sleep disorders and circadian rhythms abnormalities.

## Subjects and Methods

### Ethics statement

The local Institutional Review Boards (IRB) at the Institut Pasteur in Paris (France) approved the study. Methods were performed in accordance with the relevant guidelines and regulations. Written informed consents were obtained from all participants. For the patients who were unable to consent for themselves, a parent or legal guardian consented to the study on their behalf.

### Subjects and clinical evaluations

Clinical evaluations of patients with ASD, their relatives and control subjects have been detailed previously^10^. All the subjects were recruited into the Paris Autism Research International Sib-pair (PARIS) study. The ASD diagnosis was based on clinical expert assessment including the Autism Diagnostic Interview – Revised (ADI-R)^45^ and the Autism Diagnostic Observation Schedule (ADOS)^46^. Intellectual Quotient (IQ) was measured using an age-appropriate Wechsler scale. For the most severe and/or non-verbal patients, the Raven’s Standard Progressive Matrices and the Peabody Picture Vocabulary test were used.

### Biochemical measurements

Blood samples were collected in the morning, between 8:30 and 10:30 as described previously^10,14^. Subjects were asked to avoid food with high content of tryptophan and/or serotonin two days before blood sampling and individuals receiving exogenous melatonin were not included in this study. Melatonin was measured in plasma using a radio-immunoassay (RK-MEL, Bühlmann, Switzerland) according to the manufacturer’s instructions. Whole-blood serotonin (5-hydroxytryptamine, 5-HT) was measured by high-performance liquid chromatography^47^. NAS, as well as enzyme activities of AANAT and ASMT were determined in platelets by radio-enzymology^48^. Patients who were receiving melatonin for treatment of sleep disorders were not included in the analyses. Only pedigrees with both parents and at least one child were included in the following analyses. The overlap between the biochemical data for serotonin, AANAT, NAS, ASMT and melatonin is illustrated in Supplementary Fig. S2. A detailed study of the differences between groups has been published elsewhere^10,15^.

### Statistical methods

Data management and graphs were performed using JMP Pro 12 (SAS, USA) and R software^49^. Because some of the studied traits were not normally distributed, nonparametric statistical tests (Wilcoxon rank-sum test) were preferred to compare groups of individuals. For familial correlation and heritability analyses, serotonin, ASMT and melatonin values were logarithmically transformed in order to conform more closely to a normal distribution (Supplementary Fig. S3). Since melatonin and serotonin are known to be age and sex dependent^50–53^, they were included in the models using residuals from regression models obtained with the program Sequential Oligogenic Linkage Analysis Routines (SOLAR) 7.2.5 (Southern Foundation for Biomedical Research, San Antonio, TX, USA)^54,55^, when their effect as covariates (age, sex) were significant at the threshold of P < 0.1. Individuals at more than three standard deviations (SD) from the mean were considered as outliers and were removed. Depending on the biochemical trait and the subgroup considered, the number of outliers was always very low, ranging from 0 to 7 (Supplementary Table S3). A total of 717 individuals (182 ASD patients and 535 relatives) were included in this study. There were 185 families, including from three to nine relatives (Supplementary Table S4): 68 trios, 87 quartets, 22 quintets, four sextets, one septet, one nonet and two extended pedigrees. For three families, biochemical measurements could be obtained for the parents and unaffected children, but not for the ASD probands and these families were included in the analyses. For each analysis, uncorrected p-values are shown, but due to multiple testing, the significance was assessed after Bonferroni correction.

### Estimation of familial correlation and heritability

Parent-offspring (P-O), sibling-sibling (S-S) and father-mother (F-M) correlations were measured for the five biochemical traits using the Family Correlations (FCOR) module of the Statistical Analysis for Genetic Epidemiology (S.A.G.E.) 6.3 software package (Human Genetic Analysis Resource, Case Western Reserve University, Cleveland, OH, USA)^56^. For each relative pair types available in the sample pedigrees, FCOR calculates sibling-sibling (S-S), parent-offspring (P-O) and father-mother (F-M) familial correlations with their asymptotic standard errors. S.A.G.E then tests the difference of the correlation coefficients from 0 using Fisher’s z-transformation.

Narrow sense heritability was estimated using the variance component-based program SOLAR 7.2.5 with a maximum likelihood estimation method and polygenic models. When the effect of the covariates (age, sex) was significant (P < 0.1), relative proportion of variance explained by known covariates was estimated and they were included in the model.

SOLAR can extend the variance component model to a bivariate analysis that maximizes the model for two dependent traits^57^. The trait pairs included all pairwise combinations and the same covariates (age and sex) significant at the threshold of P < 0.1 were used. Three parameters were estimated for each pair of traits: the additive genetic correlation (ρ_G_), the shared environmental correlation (ρ_E_) and the total phenotypic correlation (ρ_P_) (Supplementary Note).

Familial correlations and univariate heritability analyses were first performed on all individuals, including parents and their affected children as well as their unaffected children (Supplementary Fig. S4). Secondly, stratified analyses were conducted on parents with their affected children and on parents with their unaffected children (Supplementary Fig. S4).

## Electronic supplementary material

Supplementary Information
