# Supplementary material for: Heritability of the melatonin synthesis variability in autism spectrum disorders
Source: Sci Rep. 2017 Dec 18;7:17746. doi: 10.1038/s41598-017-18016-3 (PMC5735101; doi:10.1038/s41598-017-18016-3)
Supplement: Supplementary file 1 — Supplementary Information [file 41598_2017_18016_MOESM1_ESM.pdf]

Supplementary Information

Heritability of the melatonin synthesis variability in autism spectrum disorders

Marion Benabou[1,2,3,4], Thomas Rolland[1,2,4], Claire S Leblond[1,2,4], Gaël A Millot[5], Guillaume Huguet[1,2,4], Richard Delorme[1,2,4,6,7], Marion Leboyer[7,8,9], Cécile Pagan[3,10], Jacques Callebert[3,10], Erik Maronde[11], Thomas Bourgeron[1,2,4,7]

Supplementary Note: Heritability estimation methods — Page 2

Supplementary Figure S1: Workflow of the present study for familial correlation and heritability analysis of the melatonin synthesis pathway in families with ASD — Page 3

Supplementary Figure S2: Overlap of the biochemical traits of the melatonin synthesis pathway investigated in the study cohort — Page 4

Supplementary Figure S3: Distribution of blood serotonin (nM), platelet ASMT activity (pmol/10^9 platelets/30 min), platelet NAS (nmol/10^9 platelets), platelet AANAT activity (pmol/10^9 platelets/30 min) and plasma melatonin levels (nM) in the cohort used for the analyses — Page 5

Supplementary Figure S4: Example of the stratification used in families to perform the analyses considering all individuals and considering only ASD children or unaffected children — Page 6

Supplementary Table S1: Heritability estimates of the melatonin synthesis pathway — Page 7

Supplementary Table S2: Phenotypic variance due to all covariates — Page 8

Supplementary Table S3: Numbers and proportions of individuals considered as outliers in the measurements of blood serotonin, platelet AANAT activity, platelet NAS, platelet ASMT activity and plasma melatonin — Page 9

Supplementary Table S4: Description of the cohorts used for the calculation of the familial correlations and heritability (h^2) — Page 10

Supplementary Note. Heritability estimation methods.

Narrow sense heritability of a quantitative trait can be estimated using the maximum likelihood method implemented in the variance component program SOLAR. The variance-components model assumes that the phenotypic variance of the trait can be partitioned into genetic and environmental components. Heritability is estimated as the ratio of genetic variance to total phenotypic variance using a maximum likelihood method, applied to a mixed-effects model that incorporates fixed effects for known covariates and variance components for genetic effects. The significance of heritability estimate is assessed using a comparison of the polygenic model to a sporadic model in which the additive genetic effect is constrained to zero.

Bivariate heritability analyses using SOLAR allow the estimation of three parameters for each pair of traits: the additive genetic correlation (ρ_G), the shared environmental correlation (ρ_E) and the total phenotypic correlation (ρ_P). If h_1^2 and h_2^2 are heritability estimates of trait 1 and 2 respectively, the estimate of ρ_P between two traits is obtained using the following equation:

$$\rho_P = [\ \sqrt{(h_1^2)}\ \sqrt{(h_2^2)}\ \rho_G\ ] + [\ \sqrt{(1-h_1^2)}\ \sqrt{(1-h_2^2)}\ \rho_E\ ]$$

These correlations values range from -1.0 to 1.0. The ρ_G and ρ_E values indicate whether the phenotypic correlation observed between two traits is due to genetic, environmental or a combination of both factors. A ρ_G significantly different from 0 indicates that the phenotypic variance of both traits is influenced by the same genetic factors. A ρ_G significantly different from 1 indicates that there are not only common genes with pleiotropic effect but also unique genetic contributions to each trait. A positive correlation implies that both quantitative traits vary in the same way in response to genetic factors, whereas a negative correlation can be observed when a trait increases while the other decreases. The ρ_E interpretation is the same as ρ_G but in terms of traits response to environmental factors.

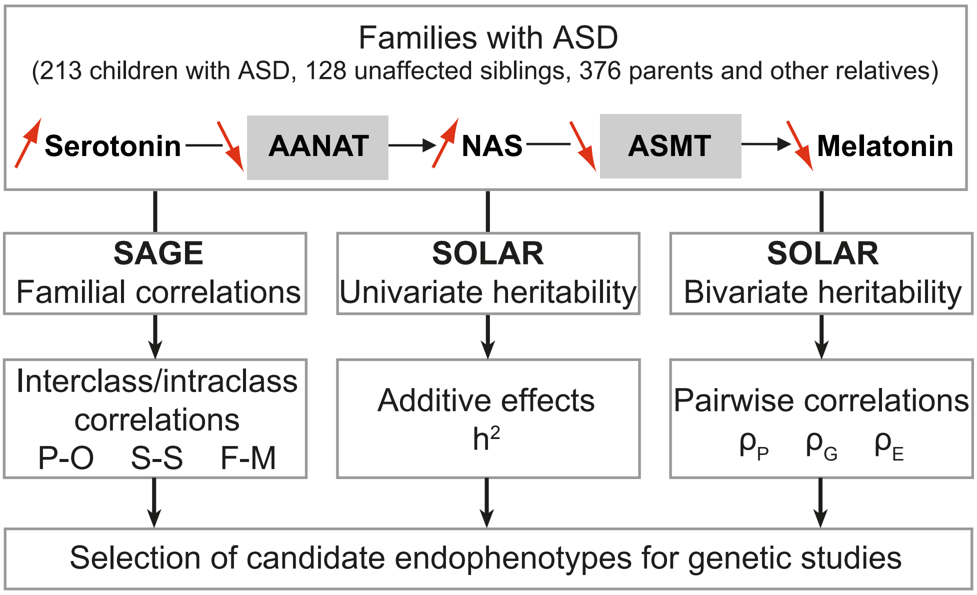

Supplementary Figure S1. Workflow of the present study for familial correlation and heritability analysis of the melatonin synthesis pathway in families with ASD. Others relatives include: 6 affected parents, 4 grandparents and 2 affected uncles. Red arrows represent biochemical anomalies reported in patients with ASD compared with controls. P-O, parent-offspring correlation; S-S, sibling-sibling correlation; F-M, father-mother correlation; h^2, narrow sense heritability estimate; ρ_G, pairwise genetic correlation; ρ_E, pairwise environmental correlation; ρ_P, pairwise phenotypic correlation.

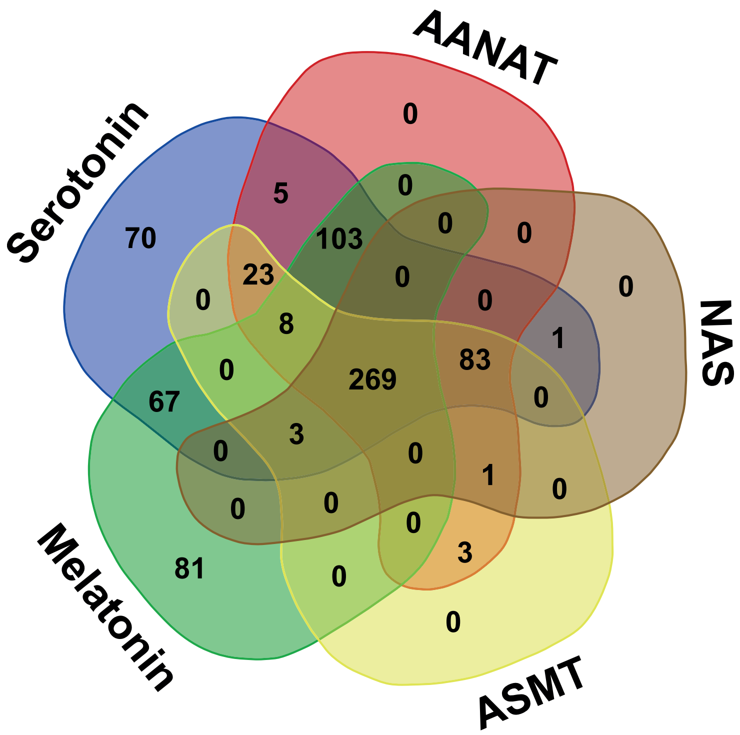

Supplementary Figure S2. Overlap of the biochemical traits of the melatonin synthesis pathway investigated in the study cohort. The numbers correspond to the individuals analyzed in this study, including only families with both parents and at least one child investigated.

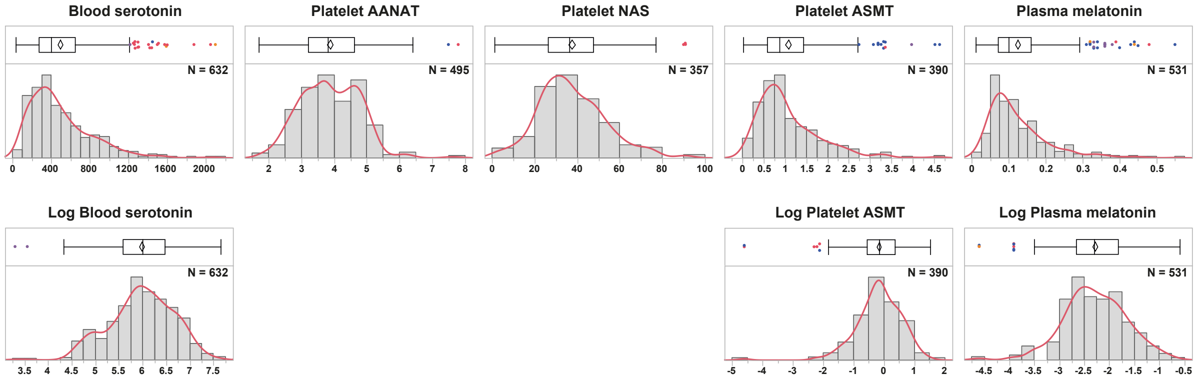

Supplementary Figure S3. Distribution of blood serotonin (nM), platelet ASMT activity (pmol/10^9 platelets/30 min), platelet NAS (nmol/10^9 platelets), platelet AANAT activity (pmol/10^9 platelets/30 min) and plasma melatonin levels (nM) in the cohort used for the analyses (excluding families for which biochemical values were not available for both parents). The distributions were represented by a boxplot and a histogram. The extreme values for the boxplot were indicated in red, blue, orange, and purple for the patients, parents, affected siblings, and unaffected siblings, respectively. Ordinate axis, proportions; red line, density curve; diamond-shape, mean and confidence interval; N, sample size.

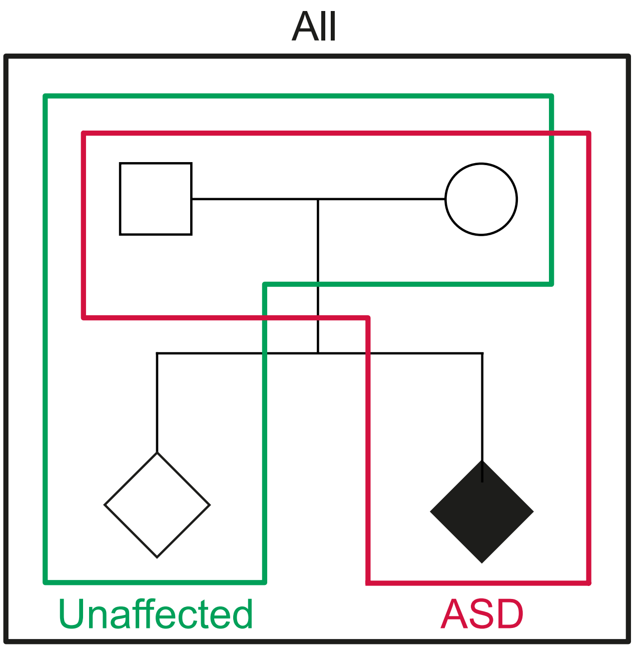

Supplementary Figure S4. Stratification used in families to perform the analyses considering all individuals and considering only ASD children or unaffected children.

Data	Group	N	h^2 (SE)	P-value
**Serotonin**	[b] All	632	0.31 (0.078)	**1.90×10^{-5}**
	[a] ASD	515	0.21 (0.095)	1.26×10^{-2}
	[a] Unaffected	275	0.43 (0.13)	**7.54×10^{-4}**
**AANAT**	[b] All	495	0.34 (0.077)	**1.40×10^{-6}**
	[b] ASD	423	0.32 (0.094)	**3.15×10^{-4}**
	[c] Unaffected	178	0.47 (0.12)	**1.92×10^{-4}**
**NAS**	[a] All	357	0.72 (0.090)	**3.52×10^{-12}**
	[a] ASD	297	0.68 (0.095)	**4.27×10^{-9}**
	[c] Unaffected	127	0.69 (0.16)	**2.01×10^{-4}**
**ASMT**	[a] All	390	0.59 (0.097)	**5.44×10^{-9}**
	[a] ASD	334	0.51 (0.11)	**1.40×10^{-5}**
	[a] Unaffected	134	0.74 (0.14)	**2.29×10^{-5}**
**Melatonin**	[a] All	531	0.22 (0.071)	**2.63×10^{-4}**
	[b] ASD	423	0.088 (0.091)	0.16
	[a] Unaffected	242	0.40 (0.11)	**1.26×10^{-4}**

Supplementary Table S1. Heritability estimates of the melatonin synthesis pathway. Significant p-values after Bonferroni correction are indicated in bold: 15 tests were performed, p-values $< 3.33 \times 10^{-3}$ (0.05/15) were considered as significant. N pairs, sample size; SE, Standard Error; [a] age was included in the model as a covariate; [b] age and sex were included in the model as covariates; [c] no covariates were included in the model.

Phenotype	Group	p-value age*	p-value sex*	Proportion of phenotypic variance due to covariates
**Serotonin**	All	**1.30×10^{-9}**	**0.083**	0.055
	ASD	**4.38×10^{-12}**	0.42	0.091
	Unaffected	**0.091**	0.80	0.012
**AANAT**	All	**0.0029**	**0.0080**	0.029
	ASD	**0.00047**	**0.081**	0.032
	Unaffected	0.41	0.63	NA
**NAS**	All	**1.77×10^{-11}**	0.16	0.049
	ASD	**2.07×10^{-13}**	0.75	0.12
	Unaffected	0.25	0.29	NA
**ASMT**	All	**4.21×10^{-13}**	0.85	0.072
	ASD	**7.56×10^{-15}**	0.54	0.13
	Unaffected	**0.032**	0.20	0.016
**Melatonin**	All	**6.59×10^{-8}**	0.58	0.045
	ASD	**1.63×10^{-11}**	**0.099**	0.10
	Unaffected	**0.015**	0.45	0.021

Supplementary Table S2. Phenotypic variance due to all covariates.

*Covariates (age, sex) with $P < 0.1$ were included in the models for familial correlations and narrow sense heritability estimation. NA, not applicable.

	Serotonin	AANAT	NAS	ASMT	Melatonin
All: nb outliers (%)	4/636 (0.63)	3/498 (0.60)	5/362 (1.38)	3/393 (0.76)	6/537 (1.12)
Unaffected: nb outliers (%)	6/281 (2.14)	0/178 (0.00)	7/134 (5.22)	0/134 (0.00)	3/245 (1.22)
ASD: nb outliers (%)	0/515 (0.00)	3/426 (0.70)	7/304 (2.30)	3/337 (0.89)	7/430 (1.63)

Supplementary Table S3. Numbers and proportions of individuals considered as outliers in the measurements of blood serotonin, platelet AANAT activity, platelet NAS, platelet ASMT activity and plasma melatonin. Individuals with values exceeding 3 standard deviations from the mean were considered as outliers and were removed from the analyses. nb outliers, number of outliers.

	Status	Samples analyzed		
		h^2 all	h^2 ASD	h^2 unaffected
Individuals	ASD	182	182	0
	Parents	364	358	146
	Unaffected Siblings	128	0	96
	Affected siblings	31	31	0
	Others*	12	12	0
	Total	717	583	242
Pedigrees	3 subjects	68	151	58
	4 subjects	87	28	10
	≥ 5 subjects	30	3	5
	Total nb of pedigrees	185	182	73

Supplementary Table S4. Description of the cohorts used for the calculation of the familial correlations and heritability (h^2).

*Others include: 6 affected parents, 4 grandparents and 2 affected uncles.
